# Spatial Memory and Blindness: The Role of Visual Loss on the Exploration and Memorization of Spatialized Sounds

**DOI:** 10.3389/fpsyg.2022.784188

**Published:** 2022-05-24

**Authors:** Walter Setti, Luigi F. Cuturi, Elena Cocchi, Monica Gori

**Affiliations:** ^1^Unit for Visually Impaired People (U-VIP), Italian Institute of Technology, Genoa, Italy; ^2^Istituto David Chiossone, Genoa, Italy

**Keywords:** audio-spatial skills, blindness, development, working memory, user-friendly technologies, acoustic perception

## Abstract

Spatial memory relies on encoding, storing, and retrieval of knowledge about objects’ positions in their surrounding environment. Blind people have to rely on sensory modalities other than vision to memorize items that are spatially displaced, however, to date, very little is known about the influence of early visual deprivation on a person’s ability to remember and process sound locations. To fill this gap, we tested sighted and congenitally blind adults and adolescents in an audio-spatial memory task inspired by the classical card game “Memory.” In this research, subjects (blind, *n* = 12; sighted, *n* = 12) had to find pairs among sounds (i.e., animal calls) displaced on an audio-tactile device composed of loudspeakers covered by tactile sensors. To accomplish this task, participants had to remember the spatialized sounds’ position and develop a proper mental spatial representation of their locations. The test was divided into two experimental conditions of increasing difficulty dependent on the number of sounds to be remembered (8 vs. 24). Results showed that sighted participants outperformed blind participants in both conditions. Findings were discussed considering the crucial role of visual experience in properly manipulating auditory spatial representations, particularly in relation to the ability to explore complex acoustic configurations.

## Highlights

- A novel task, the Audio-Memory, presented in the form of a game to evaluate audio-spatial memory abilities in sighted and blind individuals.
- Sighted outperformed the blind participants.
- Blind people encounter limitations ascribed to congenital blindness in processing auditory spatial representations and exploring complex acoustic configurations.

## Introduction

In everyday life, abilities such as comprehension, reasoning, or learning are achieved through memory processes that allow the human brain to retain spatial and non-spatial information. The cognitive system devoted to the temporary storage and manipulation of information is the working memory system (WM) (Palmer, 2000). Historically, the most supported model of WM was proposed by Baddeley (1992), who divided WM into three separate subsystems: the central executive component (involved in high-order cognitive functions), the phonological loop and the visuo-spatial sketchpad (VSSP) that are used for the storage and processing of verbal and visuo-spatial information, respectively. Logie (1995) proposed an additional division of the VSSP into two subcomponents: the “inner scribe,” which refers to spatial components of information, and the “visual cache” for processing visual features of objects. One of the main functions ascribed to the VSSP of WM is mental imagery, a cognitive function that leads to internal representations (Cornoldi and Vecchi, 2003) of the objects composing the surrounding space. This function corresponds to a quasi-perceptual experience occurring in the absence of actual stimuli for the relevant perception (Kosslyn, 1980; Finke and Freyd, 1989; Rinck and Denis, 2004). Mental imagery is directly involved in cognitive functions such as learning (Yates, 1966), problem-solving, reasoning (Féry, 2003) and original and creative thought (LeBoutillier and Marks, 2003). The nature of these representations has long been the subject of research and debate. Kosslyn’s theory (Kosslyn, 1980), the most supported in this context, posits that mental images are “picture-like” representations, as confirmed by studies involving mental rotation and mental scanning of haptic spatial layouts (Farah et al., 1988; Vingerhoets et al., 2002). Although Kosslyn’s initial theory assumed that imagery processes partially overlap with perceptual mechanisms, evidence has shown that imagery cannot be equated with visual perception (Cornoldi and Vecchi, 2003). Visual mental images are not mere copies of visual input but rather the end product of a series of constructive processes based on memory retrieval mechanisms (Pietrini et al., 2004). Therefore, visuo-spatial mental imagery can originate from different sensory and perceptual inputs (e.g., visual, haptic, acoustic and verbal) (Cornoldi and Vecchi, 2003). Supporting this view, neuroimaging and electrophysiological studies generally indicate that the maintenance of information in spatial WM is not modality–specific and does not strictly depend on the encoding sensory modality (Lehnert and Zimmer, 2006, 2008).

Studies with congenitally blind individuals can provide fundamental insights into the role of vision in spatial memory abilities within the imagery debate. Visually impaired individuals can generate and manipulate mental images through long-term memory, haptic exploration, or verbal description (Zimler and Keenan, 1983; Lederman and Klatzky, 1990; Carreiras and Codina, 1992). Visual features such as dimension, shape, or texture can be perceived through touch and conveyed in internal images. Thus, the absence of sight does not impede an efficient visuospatial system functioning.

Blind individuals show deficits in memory tasks requiring large sequences of mental manipulation of stored information, namely active memory tasks (Vecchi et al., 1995, 2004; Vecchi, 1998). When the experimental demand requires only maintaining small amounts of information instead (i.e., passive memory tasks), their abilities usually do not differ significantly from sighted people (Cornoldi and Vecchi, 2003; Setti et al., 2018, 2019). Nevertheless, blind individuals might also show limitations when only passive memory processes are involved, such as memorizing 2D spatial layouts (Vecchi, 1998). In fact, vision remains the preferred sensory modality that facilitates the accomplishment of visuospatial working memory tasks, especially when great demands on memory are required.

Blind individuals do show limitations when asked to continuously process the mental image of a previously learned spatial layout (Juurmaa and Lehtinen-Railo, 1994) or when performance can be enhanced through active manipulation of spatial information (Setti et al., 2018). Moreover, blind individuals encounter difficulties using perspective in mental representations (Arditi and Dacorogna, 1988) and in elaborating the third dimension when learning a haptic spatial layout (Vecchi, 1998). When increasing the number of items to be actively processed, thus increasing task demand, blind individuals demonstrate inferior performance compared to sighted individuals (Vecchi, 1998; Vanlierde and Wanet-Defalque, 2004; Cattaneo et al., 2008). Vision is the best sensory modality through which the brain processes several items simultaneously (De Beni and Cornoldi, 1988), and as such, lack of vision impacts this ability (Cornoldi and Vecchi, 2003).

There is evidence that vision plays a crucial role in guiding the maturation of spatial cognition (Hart and Moore, 2017). In early visual deprivation, the remaining intact sensory modalities are recruited to process spatial information. In tactile tasks such as object recognition and immediate hand-pointing localization, visually impaired individuals perform as well or better than sighted controls (Morrongiello et al., 1994; Rossetti et al., 1996; Sunanto and Nakata, 1998). In the auditory processing of space, blind individuals exhibit enhanced abilities for azimuthal localization (King and Parsons, 1999; Roder et al., 1999; Gougoux et al., 2004; Doucet et al., 2005) and relative distance discrimination (Voss et al., 2004; Kolarik et al., 2013). At the same time, blind people show significant impairments for auditory spatial tasks such as vertical localization, absolute distance discrimination and spatial bisection (Zwiers et al., 2001; Lewald, 2002; Gori et al., 2014). Thus, early visual deprivation affects performance in tasks requiring a complex representation of space and it has been argued that these deficits reflect a lack of visual calibration over touch and audition in processing spatial information (Gori et al., 2014). According to the cross-sensory calibration hypothesis, vision calibrates the other sensory modalities to process spatial information. In other words, the brain learns from vision how to evaluate objects’ orientation and proprioceptive position through alternate sensory modalities such as audition and touch (Cappagli et al., 2017; Cuturi et al., 2017). Another explanation for the decreased performance of visually impaired individuals in complex spatial tasks is that it originates from a compromised spatial memory. This hypothesis is supported by studies demonstrating that blind children have limitations in spatial recall (Millar, 1976) and the simultaneous processing of multiple representations (Puspitawati et al., 2014). These results do not suggest that mnemonic skills in general are impaired in blind individuals. Blind individuals ably perform temporal tasks that require participants to understand and remember the temporal order of sound presentations (Vercillo et al., 2016). They show limitations only in tasks requiring complex spatial judgments (Bertonati et al., 2020) where the spatial presentation of stimuli position is fundamental to accomplish the task (Gori et al., 2014).

Lack of visual experience may also lead to the differential use of spatial reference frames to encode the information to be memorized. The two main frames of reference used to represent the location of entities in space are egocentric and allocentric (Ruggiero and Iachini, 2010). The first defines locations of items in the surrounding environment from the observer’s perspective and in relation to observer’s position. Conversely, allocentric reference frames encode spatial information by considering external landmarks and spatial relationships among the items regardless of observer’s position. In the context of spatial memory, previous research has demonstrated that spatial information is organized according to reference frames defined by the layout itself and not by egocentric experience (Mou and McNamara, 2002). Depending on the task to be accomplished, sighted individuals can rely on allocentric frames of reference to orient themselves or to represent and memorize spatial information (Ruggiero et al., 2012; Pasqualotto et al., 2013; Iachini et al., 2014). In contrast, early visual deprivation results in significant impairments in tasks that require an allocentric representation of space (Thinus-Blanc and Gaunet, 1997; Arnold et al., 2013; Gori et al., 2014).

Most research investigating spatial memory in vision loss has been carried out in the haptic domain (Vecchi et al., 1995; Bonino et al., 2008, 2015). Although haptic information plays a substantial role in processing objects proximal to the observer, auditory information allows visually impaired individuals to process surrounding information, including items that are not directly reachable by the observer. For instance, in spatial navigation, sensory substitution devices can aid the ability to build mental maps by integrating auditory and self-motion information (Jicol et al., 2020). In the study, visually impaired individuals could take advantage of visual information converted to acoustic cues more efficiently than sighted participants when performing both egocentric and allocentric navigation tasks, thus indicating that multisensory cueing of space may reduce blindness-related deficits. Conversely, in the context of spatial memory, Setti et al. (2018) demonstrated that congenitally blind individuals show limitations when asked to manipulate spatial information in recalling sequences of spatialized sounds in the acoustic sensory domain.

**FIGURE 1:**
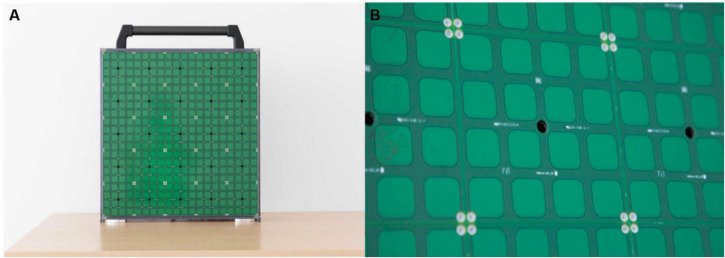
ARENA2D at two levels of detail. **(A)** Presents the device. ARENA2D is a vertical surface (50 × 50 cm) composed of 25 haptic blocks, each with a loudspeaker in the center, arranged in the form of a matrix. **(B)** Shows a single haptic block in detail. The black hole is the speaker from which the sound is emitted. The blocks are covered by 16 (4 × 4 matrix) tactile sensors (2 × 2 cm^2^) that register the position of each touch.

To deeply investigate audio-spatial memory and exploration strategies in blindness, we focus on comparing how blind and sighted individuals construct and manipulate a dynamic auditory structure in a spatial memory task. In the context of this study, the term “dynamic” refers to a spatial structure whose configuration needs to be continuously updated. This aspect reflects everyday life experiences where surrounding acoustic information constantly changes and provides a fundamental sensory cue for blind people to represent the surrounding environment. We tested ability to hold spatialized sounds in memory and update the mental representation of their locations. With this goal in mind, inspiration was taken from the card game “Memory,” which works on attention, memory and concentration (da Cunha et al., 2016). By playing this card game, it is possible to improve concentration, train short-term memory, strengthen associations between concepts, and classify objects grouped by similar traits. In its original form, the game consists of covered cards lying on a table, and the goal is to find pairs among the cards. We adapted the “Memory” game to the auditory domain by employing a vertical array of speakers named ARENA2D (see Figure 1). We call this novel task “Audio-Memory.” Participants were required to match sounds that were spatially displaced over the audio-tactile device. To investigate the impact of memory load on performance, we designed two experimental conditions by increasing the number of sounds paired, using four pairs in the 4-pair condition and 12 pairs in the 12-pair condition (named 4-pair and 12-pair conditions, respectively, for brevity). With the Audio-Memory task, we addressed the following research questions:

1. To what extent does early visual deprivation influence audio-spatial memory skills?
2. What is the exploration strategy used by the two groups when asked to explore a complex auditory structure to construct a spatial representation of sound dispositions?

Since vision is crucial for spatial processing (Alais and Burr, 2004; Burr et al., 2009; Hart and Moore, 2017) we hypothesized that a lack of visual experience would affect audio-spatial memory skills in blind individuals. Due to the great cognitive load required to manipulate spatial information and the difficulties in using the spatial relations among the sounds, we expected sighted to outperform blind participants. Furthermore, in the context of spatial exploration, we hypothesized that congenitally blind participants would explore the layout of speakers differently compared to the sighted group. Considering how the lack of visual experience affects spatial processing in blind individuals, we expected them to show a slower and more sequential exploration of the spatially distributed items compared to sighted participants.

## Results

**FIGURE 2:**
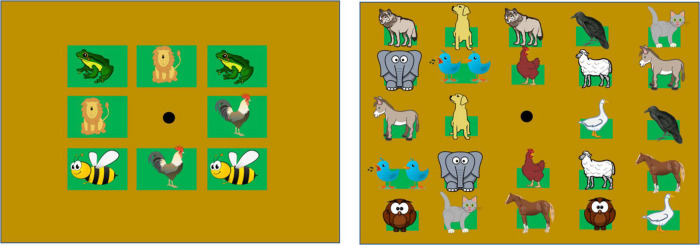
Grids used in experimental conditions. The two grids differ in the size of the apertures for each auditory item. The apertures on the grids represented in the left column are 10 cm × 10 cm, equal to the haptic block size. The apertures on the grids represented in the right panel are 4 cm × 4 cm. Depicted animals placed inside the squares, refer to the position of the animal calls in each grid (images downloaded from a royalty-free website, https://publicdomainvectors.org/). The black dot at the center indicates the speaker emitting feedback sounds.

We tested blind and sighted adolescents and adults. All groups performed two experimental tasks, consisting of four and twelve pairs of sounds to be matched (Figure 2). To deliver sounds, we used ARENA2D, a 5-by-5 matrix of speakers covered by tactile sensors, each constituting a haptic block (Ahmad et al., 2019; Setti et al., 2019; Figure 1).

**FIGURE 3:**
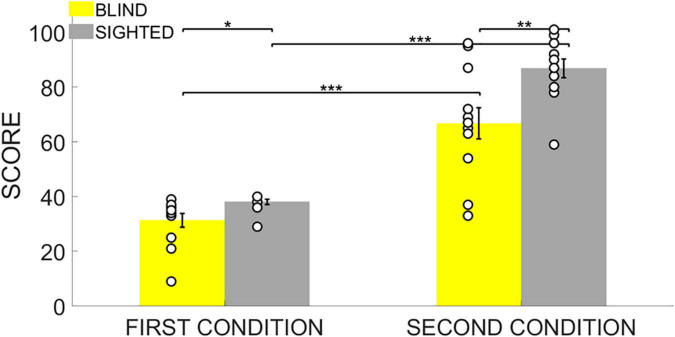
Score. Data are presented as mean and standard error for each group. The white circles on the bars represent the individual data. The Score reached by the participants was lower in the 4-pair condition and the sighted outperformed the blind group in both experimental conditions. * indicates *p* < 0.05, ** indicates *p* < 0.01, *** indicates *p* < 0.001.

**FIGURE 4:**
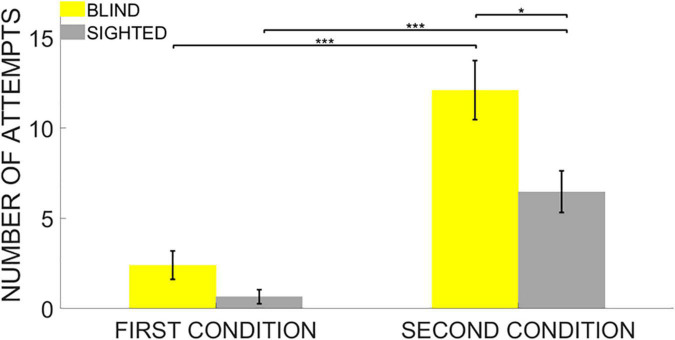
Number of Attempts. Data are presented as mean and standard error for each group. The white circles on the bars represent the individual data. Even though both groups needed more attempts to end the task in the 4-pair compared to the 12-pair conditions, the sighted group needed fewer attempts to pair the items once their locations have been discovered on ARENA2D but only in the second condition. No significant difference between the groups was found in the first condition instead. * indicates *p* < 0.05, *** indicates *p* < 0.001.

**FIGURE 5:**
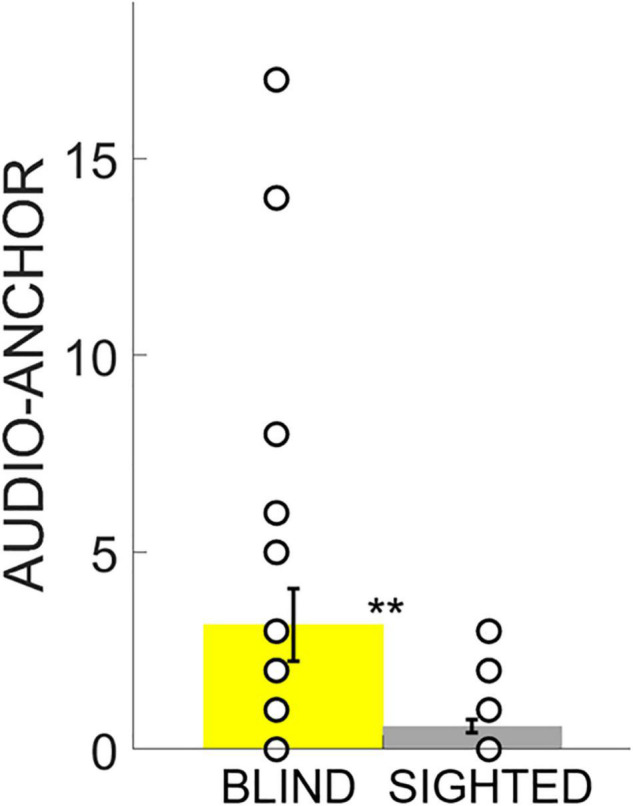
Audio-Anchor. Data are presented as the mean and standard error. The white circles on the bars represent the individual data. The blind group relied more on the use of the Audio-Anchor regardless of the experimental condition. ** indicates *p* < 0.01.

Participants sat facing ARENA2D at a distance of 30 cm. Memory performance was evaluated using the Score reached at the end of the test and the Number of attempts to pair the two stimuli once positions were discovered. Finally, we defined the Audio-Anchor index as an expression of spatial exploration strategy that measured how often participants started two consecutive attempts by touching the same haptic block (see “Data Analysis” for further details). Statistical analyses were conducted using RStudio (Version 1.1.463) and data are shown as means and standard error (Figures 3, 4, 5). Given the small sample size, statistical analyses were conducted with non-parametric tests based on permutations. We first ran a non-parametric MANCOVA (*adonis2()* R function) with Score, Number of attempts and Audio-Anchor as dependent variables, *Group* (either blind or sighted) as between-subject, *Difficulty* (either easy or hard) as within-subject and *Age* (the age of the participants in decimal number of years) as a covariate. Follow-up analyses were conducted only for the significant interactions with ANOVAs based on permutations (*aovp()* R function). Finally, *post hoc* analyses were run with unpaired Student’s *t*-tests based on permutations (*perm.t.test()* R function) and *Bonferroni* corrections were used to correct for multiple comparisons (see the Statistical Analyses section for further details).

Results of the non-parametric MANCOVA (number of permutations = 999) highlighted a significant main effect of the *Group* [*F*(1,40) = 9.124, *p* = 0.001, *η^2^* = 0.243], a significant main effect of *Difficulty* [*F*(1,40) = 72.62, *p* = 0.001, *η^2^* = 0.909] and a significant interaction *Group* * *Difficulty* [*F*(1,40) = 3.35, *p* = 0.032, *η^2^* = 0.202] but no significant main effect of *Age* [*F*(1,40) = 0.44, *p* = 0.626], nor significant interactions Group* *Age* [*F*(1,40) = 0.915, *p* = 0.367], *Age* * *Difficulty* [*F*(1,40) = 0.07 *p* = 0.939] nor *Group* * *Age* * *Difficulty* [*F*(1,40) = 1.87, *p* = 0.154]. Thus, given that the only significant interaction was *Group* * *Difficulty* we ran a follow-up non-parametric ANOVA, separately for the Score the Number of attempts and the Audio-Anchor, with *Group* as between-subject and *Difficulty* as within-subject factors and by putting together adolescents and adults in both groups. The results of MANCOVA indeed highlighted that the performance in the task were not affected by participant ages.

The ANOVA of the Score, showed a significant main effect of the *Group* (iterations = 5,000, *p* < 0.001, *η^2^* = 0.24), a significant main effect of the *Difficulty* (iterations = 5,000, *p* < 0.001, *η^2^* = 0.76) and a significant interaction *Group* * *Difficulty* (iterations = 2,131, *p* = 0.041, *η^2^* = 0.07). As expected, *post hoc* analyses first revealed that both blind and sighted participants reached a higher score in the first condition because it was easier in terms of number of stimuli to be paired (unpaired two-tailed *t*-test based on permutations: Welch’s *t* = 5.66, *p* < 0.001, Cohen’s *d* = 1.01, Welch’s *t* = 2.49, *p* < 0.001, Cohen’s *d* = 5.67 for both the blind and sighted groups, respectively). Interestingly, we found that, compared to blind participants, sighted participants reached a higher Score in both the 4-pair (unpaired two-tailed *t*-test based on permutations: Welch’s *t* = 2.50, *p* = 0.031, Cohen’s *d* = 1.02) and 12-pair condition (unpaired two-tailed *t*-test based on permutations: Welch’s *t* = 3.02, *p* = 0.01, Cohen’s *d* = 1.23) compared to the blind group (Figure 3).

The results of the ANOVA on the Number of attempts also highlighted a significant main effect of the *Group* (iterations = 5,000, *p* < 0.001, *η^2^* = 0.21), a significant main effect of the *Difficulty* (iterations = 5,000, *p* < 0.001, *η^2^* = 0.53) and a significant interaction *Group* * *Difficulty* (iterations = 2,329, *p* = 0.04, *η^2^* = 0.07). As expected, also in this case, *post hoc* analyses first revealed that both blind and sighted participants needed more attempts to end the task in the second condition due to the greater number of sounds to be paired (unpaired two-tailed *t*-test based on permutations: Welch’s *t* = 5.35, *p* < 0.001, Cohen’s *d* = 2.18, Welch’s *t* = 4.79, *p* < 0.001, Cohen’s *d* = 1.96 for both the blind and sighted groups, respectively).

*Post hoc* analyses revealed no significant difference between the two groups in the Number of Attempts required to end the task in the first condition (unpaired two-tailed *t*-test based on permutations: Welch’s *t* = 1.98, *p* = 0.21). Nevertheless, the blind group needed more attempts to end the task in the second condition compared to the sighted group (unpaired two-tailed *t*-test based on permutations: Welch’s *t* = 2.815, *p* = 0.035, Cohen’s *d* = 1.15). Results for Number of Attempts confirmed that blind participants did not hold item locations in memory as efficiently as sighted participants, especially when the cognitive load of the task increased in the second condition (Figure 4).

Finally, we used the Audio-Anchor index to compare the strategies of the two groups when exploring the device and completing the task. The results of the ANOVA highlighted a main effect of the *Group* (iterations = 5,000, *p* < 0.001, *η^2^* = 0.16) and of the *Difficulty* (iterations = 4,735, *p* < 0.001, *η^2^* = 0.09), but no significant interaction between the two (iterations = 238, *p* = 0.3). *Post hoc* analysis revealed no significant difference between the first and second conditions in the tendency to use the Audio-Anchor as an exploration strategy, regardless of the experimental group (unpaired two-tailed *t*-test based on permutations: Welch’s *t* = 1.88, *p* = 0.063). However, the analyses indicated that blind participants were more prone to using this exploratory strategy in both the first and second conditions compared to the sighted group (unpaired two-tailed *t*-test based on permutations: Welch’s *t* = 2.76, *p* < 0.01, Cohen’s *d* = 0.56) (Figure 5).

## Discussion

This research investigated how blind people memorize, learn, and process acoustic spatial information and complex auditory spatial structures. To this aim, we adapted the card game “Memory” to be administered with blind and sighted participants in the form of an experimental paradigm with spatialized acoustic items instead of cards. Participants were asked to pair animal calls that were spatially displaced in two experimental conditions of increasing difficulty (the first one with eight and the second one with 24 items). In comparison to sighted individuals, we hypothesized that blind participants would encounter more difficulties when asked to process complex audio-spatial representations. In support of our hypotheses, we observed that the sighted group outperformed the blind group in both conditions by reaching a higher Score (Figure 3).

Furthermore, in the more difficult condition with twelve pairs to be matched, blind subjects needed more attempts to proceed with the task and returned more times on the same haptic blocks than the sighted group (Figure 4). This suggests that lack of vision may lead to difficulties in integrating the spatial positions of sounds into a coherent and functional spatial representation. Moreover, these results indicate that the absence of visual experience affects the employment of functional spatial exploration strategies in the discovery and memorization of non-visual auditory spatial structures regardless of the experimental condition (Figure 5).

Observed differences between blind and sighted participants can be related to difficulties in combining the spatial position of sound sources in a coherent and functional mental representation. The Audio-Memory indeed, requires the active manipulation of spatial information, a mental operation generally affected by congenital blindness. In the haptic modality, blind individuals can construct a mental representation of a tactile layout and remember the locations of the targets on their surface (Vecchi et al., 2005; Cattaneo et al., 2008). The same ability has been observed with acoustic items (Setti et al., 2018). Following the exploration of a complex and meaningful spatial auditory scene, when asked to recall the position of the items composing the layout one by one, blind and sighted participants performed equally well (Setti et al., 2018). Thus, after the exploration of a spatial arrangement, blind people should overall be able to recall the positions of all items composing a certain configuration (Vecchi, 1998; Cornoldi et al., 2000). Mental representations can indeed be built even in the absence of external visual inputs. In general, when the task demand is the simple memorization of items and the cognitive load imposed by the task is not high, blind and sighted individuals perform similarly (Cattaneo et al., 2008; Cattaneo and Vecchi, 2011). Nevertheless, good performance of blind individuals in spatial memory tests, even passive, strongly depends on the demands on memory and on the amount of spatial elaboration that is needed to perform the task. Visual perception is indeed the “preferred modality” in visuo-spatial working memory and previous studies highlighted that also with simple 2D patterns, blind participants performed poorly (Vecchi, 1998). In Setti et al. (2018), we found that blind participants could remember the spatial positions of the stimuli embedded in ARENA2D, the same device used in the current study. The better performance of the sighted group was only ascribed to a better use of the spatial relations among the sounds and not to the simple memorization of their locations. The same pattern of results was confirmed in another work that relied on an acoustic virtual reality system (Setti et al., 2021a), where the blind group could easily remember sounds’ locations after a spatial exploration of the virtual environment. In the current study, the task required participants to generate a mental spatial image of the audio spatial structure of the items to be remembered. Thus, it is a more complex task than the simple memorization of items’ locations. Specifically, participants were required to memorize and manipulate the mental representation of a complex and dynamic acoustic layout. The dynamic aspect requires a continuous updating process occur while performing the task by maintaining locations in memory. When a new item is discovered, participants must remember its location and, at the same time, update the spatial representation of the scene by adding the uncovered sound’s position. Conversely, when two items were paired, their sites were covered by cardboard squares, thus removing them from the represented scene. These processes progressively increased the cognitive load imposed by the experimental paradigm as the subject proceeded toward the end of the task. In this sense, the differences observed between blind and sighted participants may reflect the greater need for blind individuals to use executive functions affected by increasing cognitive load. Along these lines, De Beni and Cornoldi (1988) observed that congenitally blind individuals experience more difficulties in spatial WM tasks that have high memory demands than sighted individuals.

Previous research has also shown that blind people have difficulty dealing with multiple haptic stimuli presented simultaneously (Vecchi et al., 1995). Our results lead to similar conclusions in the context of spatial memory of acoustic items. In the study presented here, we observed that blind participants tended to use an audio-anchor strategy to explore the audio-spatial structure more than sighted participants (Figure 5). In other words, blind participants were more likely to build their spatial representation of the auditory structure piece-by-piece by referring all spatial locations to a previously explored position on ARENA2D. Thus, in comparison to sighted, blind individuals seem to be less able to organize and maintain spatialized auditory information thus suggesting that the absence of visual experience confines WM abilities to a more sequential and slower processing of spatial information (Pascual-Leone and Hamilton, 2001; Cattaneo et al., 2008; Ruggiero and Iachini, 2010). As a result of their visual experience, sighted people can better code spatial information in the form of global, externally based representations (Cornoldi et al., 1993; Cattaneo et al., 2008). In line with the calibration theory on the development of multisensory processing of spatial information (Gori et al., 2012), visual experience appears to be fundamental for developing a functional representation of spatial information in structured patterns (i.e., chunks). Previous research on the simultaneous manipulation of multiple stimuli suggests that visual experience is needed to acquire such ability even if stimuli are not visually conveyed (De Beni and Cornoldi, 1988). In this context, we interpret our results as evidence of the influence of visual experience in multisensory processing of simultaneous stimulation. The ability to process different sounds simultaneously and represent spatial information in the form of structured patterns may have helped sighted participants in updating the spatial representation of stimuli’s locations during the execution of the Audio-Memory task. Such ability seems to be compromised in blind participants, as expressed by their greater tendency to start consecutive trials by exploring previously discovered items. Similar to previous observations in the context of haptic spatial memory (Ruggiero and Iachini, 2010), blind individuals show limited functional strategies to process spatial information, thus suggesting that their performance required a greater involvement of executive resources compared to sighted.

Since participants generated the auditory feedback through their arm movements, spatial information emerged from sensorimotor contingencies’ coupling. In other words, to touch the sensors and emit the sound corresponding to each haptic block, the participant had first to reach the location with their arm. The movement of the arm could have been used as a cue to identify and consequently remember sound positions because the stimulus was generated after the touch. Past research showed that audition provides informative feedback on limb movement, enhancing localization skills after training (Bevilacqua et al., 2016; Cuppone et al., 2019). In the Audio-Memory task presented here, participants coupled their arm movement with spatial acoustic feedback.

Finally, independent of the presence of visual disability, we did not observe significant differences in the performance between adults and adolescents. Given that 12-year-old pre-adolescents have already reached an adult-like performance in a variety of sensory and cognitive tasks (Vuontela et al., 2003; Peterson et al., 2006; Luna, 2009; Scheller et al., 2021) we did not expect age-related differences in performance on the Audio-Memory task. Our results confirm similar age-related achievements for sighted and blind individuals but we cannot exclude that such differences might be present in a younger population tested with the Audio-Memory task. Finally, given that the task is designed in the form of a game, our experimental paradigm would be suitable to pursue studies in this direction to elucidate the influence of blindness in the development of audio-spatial memory skills. As shown here, the adaptation of a game in an experimental protocol allows the use of such a procedure with visually impaired individuals across a wide age range, including those in late childhood and potentially also with younger children. Beyond scientific settings, the Audio-Memory may be adapted for educational purposes as a tool to speed learning and development of new concepts and associations, facilitating the inclusion of visually impaired individuals in educational contexts.

## Conclusion

This study evaluated spatial and memory skills of blind and sighted individuals and their strategies for exploring complex auditory structures. Early visual deprivation affects the processing and exploration of spatial items embedded in a complex acoustic structure. With higher cognitive demands (such as those required in the 12-pair condition), blind subjects needed more attempts to update the spatial information learned during the task than sighted participants. Furthermore, blind participants relied more on the audio-anchor strategy to explore ARENA2D and build a functional, unified and constantly updated spatial representation. In line with previous findings, limitations previously observed in the haptic domain (Vecchi et al., 2005), held for the auditory modality, thus confirming the pivotal role of visual experience in the active manipulation of memorized spatial information. The current paradigm, designed in the form of a game, can be used as a starting point to define novel procedures for cognitive evaluation and rehabilitation. In addition, the Audio-Memory task might be suitable for developing multisensory training to enhance spatial representation through the coupling of auditory and proprioceptive cues. These procedures might be used in those clinical conditions where using the auditory modality can be more effective than vision, such as in the context of visual impairment or cognitive and neuropsychological impairments.

## Materials and Methods

### Participants

Twelve congenitally blind (nine females; age range: 12–52 years, mean age ± SD: 25.5 ± 15.29 years, ethnicity: Caucasian) and twelve sighted (nine females; age range: 12–54 years, mean age ± SD: 25.83 ± 15.86 years, ethnicity: Caucasian) individuals took part in the experiment. In the recruitment process, we used a broad age range because of general difficulties in recruiting congenitally blind individuals. Clinical details relative to their visual impairment are given in Table 1. Blind adults were recruited from our institute database and blind adolescents from the “Istituto David Chiossone” based in Genoa, Italy. The local health service ethics committee approved the experiments (Comitato Etico, ASL 3, Genoa, Italy). Parental or adult informed written consent for the study was obtained in all cases. All experiments were performed under The Declaration of Helsinki. None of the sighted or blind participants had any additional sensory or cognitive disabilities.

**TABLE 1:** Blind participants’ clinical details.

Participant	Gender	Age	Pathology	Residual vision
S1	M	14	Uveitis	Lights and shadows
S2	F	13	Retinopathy	Light and shadows
S3	F	12	Retinopathy of prematurity	No vision
S4	F	15	Leber’s amaurosis	No vision
S5	F	15	Cataract	No vision
S6	M	52	Retinopathy of prematurity	No vision
S7	F	30	Retinopathy of prematurity	No vision
S8	F	12	Glaucoma	Lights and shadows
S9	F	42	Leber’s amaurosis	No vision
S10	M	25	Retinitis pigmentosa	No vision
S11	F	52	Retinitis pigmentosa	No vision
S12	F	24	Retinitis pigmentosa	No vision

### Setup and Stimuli

The test was performed using a vertical array of speakers, arranged in the form of a matrix (50 × 50 × 10 cm) called ARENA2D (see Figure 1 for details) that allowed for the serial emission of spatialized sounds.

This device is comprised of 25 haptic blocks (10 × 10 cm^2^), each covered by a 4 × 4 matrix of tactile sensors (2 × 2 cm^2^ each) (see Figure 1 for details). When a tactile sensor of a haptic block is touched, touch position is registered. For each haptic block, the sound is emitted from the speaker belonging to the haptic block itself (see the black holes in Figure 1B), thus sounds are spatially distributed over the surface of ARENA2D. All blocks are connected in cascade through USB cables [see technical details in Setti et al. (2019)]. Two cardboard grids were used to allow for haptic exploration of the device while performing the task. The grids were developed in collaboration with rehabilitators from the David Chiossone Institute (Genoa, Italy). Following rehabilitators’ indications, apertures were as big as the haptic blocks in the 4-pair condition (10 × 10 cm^2^), while we used smaller apertures for the 12-pair condition (4 × 4 cm^2^) to facilitate haptic exploration and coding of each position’s device. To avoid performance being influenced by localization abilities, we chose the size of apertures to overcome auditory localization error previously observed in blind individuals, i.e., 3 cm (Cappagli et al., 2017).

The sounds chosen were distinctive animal calls to ensure ease of discrimination (Table 2). All sounds were downloaded from an online database of common licensed sounds^1^, equalized and reproduced from the speakers at the same volume. All sound clips lasted 3 s to support easy recognition by participants. Feedback sounds about the performance were emitted from the central speaker of ARENA2D (Figure 1): a “Tada!” sound when two items were matched and a recorded voice saying “NO” otherwise. At the end of the task, a jingle was played from the central speaker to make the test engaging. The sound pressure level (SPL) was maintained at 70 dB and the Root Mean Square (RMS) level was calibrated to be the same across the various signals.

**TABLE 2:** Stimuli employed in both experimental conditions.

Sound	Condition
Bee	1
Lion	1
Frog	1
Rooster	1
Wolf	2
Dog	2
Crow	2
Cat	2
Elephant	2
Birds	2
Hen	2
Sheep	2
Donkey	2
Goose	2
Owl	2
Horse	2

### Experimental Procedure

The experimental protocol was an adapted audio version of the classic card game “Memory,” designed to be performed by blind individuals. Cards were replaced with sounds (animal calls) and two experimental conditions (Figure 2) considered. In the 4-pair condition, participants searched for four pairs of identical animal calls; in the 12-pair condition, there were 12 pairs to be matched. We used different sets of animal calls for each condition. To differentiate between experimental conditions, we took advantage of two cardboard grids that differed in the number of apertures and shape (Figure 2). Overall, the 12-pair condition required increased memory load.

During the experiment, subjects sat on a chair at a distance of 30 cm from the device, whose position was adjusted to align the subject’s nose with the grid’s central aperture. None of the participants had previously interacted with ARENA2D, and the group of sighted participants entered the room already blindfolded. The experimenter guided the subject’s hands to explore the grid with eight apertures and counted them with the participants by guiding their hands over the grid and its apertures. After this phase, participants freely touched ARENA2D with both hands to familiarize themselves with the device. During the actual test, subjects were instructed to use the index finger on their dominant hand to select items and explore the device. Before starting the experiment, participants practiced with a trial session. Using the 4-pair condition grid, the experimenter guided the participant’s hand, first over two unpaired free slots (that emitted different sounds), and then over two paired items, to familiarize them with the task and the feedback sounds. After this practice session, participants listened to and identified each animal call. The experimenter confirmed that all participants recognized all animal calls. After the recognition phase, the test started with the 4-pair condition (Figure 2, left panel). Once this session had finished, the grid for the second experimental condition was placed over the device (Figure 2, right panel). Subjects explored this grid by counting the free apertures with the experimenter’s help and then exploring the device with no guidance. When the subjects were confident with the grid, the 12-pair condition started. All the subjects were instructed not to move the head through the experiment. In the case of head movements, the experimenter stopped the test to adjust participant’s head. The test did not have a fixed duration since it was self-paced. However, each session lasted 25 min on average.

### Data Analysis

In both conditions, to quantify subjects’ performance, three parameters were calculated to measure memory and exploration strategy. Details of parameters follows.

#### Score

**FIGURE 6:**
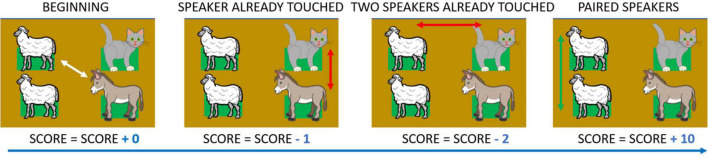
Score, example of calculation. Score is an index that decreases when participants press a panel they have previously chosen. When two blocks are touched for the first time a Score of 0 is allocated. If they have already touched one or both blocks, the Score decreases by one or two, respectively. When a pair is found the Score increases by ten. In the example, if the starting value were equal to zero, the final Score would be: 0 – 1 – 2 + 10 = 7. Depicted animals were downloaded from a royalty-free images web archive (https://publicdomainvectors.org/).

The number of touches on the same haptic block (Figure 6). The more participants touched the same haptic block, the lower their Score. This parameter quantified the overall memory performance in the test. Score was calculated as follows. For each attempt, if both haptic blocks touched were selected for the first time, the total Score was neither increased nor decreased. In fact, in this case, memory processes did not influence item choice as they were not previously discovered. If, in an attempt, only one of the two haptic blocks had already been touched, the total Score decreased by one. If both haptic blocks had already been touched in an attempt, the total Score was decreased by two. In this and the previous case, the Score was decreased to account for inferior performance in recalling the position of the previously uncovered item. When a pair was found, regardless of the number of touches per each haptic block, the total Score was increased by 10. Figure 6 shows a detailed example of how Score is calculated.

#### Number of Attempts

This quantifies how many attempts the subject required to pair two identical items once their positions had been discovered on the ARENA2D. The higher the value, the more attempts were needed to pair the sounds, and therefore, the worse the performance. This parameter quantifies the ability to maintain the spatial locations of uncovered items in memory. The Number of Attempts to pair sounds once their locations were discovered on the device was calculated for each possible pairing. For each participant, we summed up the number of trials to pair each couple of sounds (four and twelve pairs for the 4-pair and 12-pair condition, respectively). Then, we averaged these numbers and we obtained a mean number of trials to pair two sounds for each participant. Finally, these means were mediated across all the subjects.

#### Audio-Anchor

**FIGURE 7:**
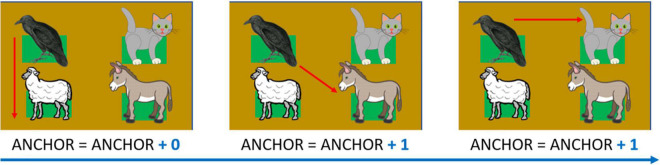
Audio-Anchor, example of calculation. The index equals zero at the beginning of the test. The index increases with more attempts starting with the same haptic block. In the presented example, the final value would be: 0 + 1 + 1 = 2. Depicted animals were downloaded from a royalty-free images web archive (https://publicdomainvectors.org/).

This index accounts for how many consecutive attempts the participant makes by starting with the same haptic block and measures exploration strategy. In other words, this index evaluates how many times the participants started consecutive attempts from the first haptic block of the last pair that they tapped. As this strategy is increasingly adopted, the index increases. For instance, suppose that the participant encounters, in an attempt, the cat meow first and the dog bark after. The Audio-anchor index would increase by one if they began the subsequent attempt again from the same cat’s meow position. The index increases until the child starts an attempt by touching a different stimulus position. As in a previous study (Setti et al., 2021b) the audio-anchor provides a measurement of how well the person construct their spatial representation of sound disposition. Thus, the greater the final index, the less the mutual relationship among the stimuli are used (see Figure 7 for details).

### Statistical Analyses

All analyses were carried out in RStudio, Version 1.1.463 (R Core Team (2020), 2020) with non-parametric tests given the small sample size. We first ran a two-way repeated measures MANCOVA based on permutations (*adonis2()* R function) using the R package “vegan” (Dixon, 2003) with Score, Number of attempts and Audio-Anchor as dependent variables, *Group* (either blind or sighted) as between-subject, *Difficulty* (either easy or hard) as within-subject and *Age* (i.e., the age of the participants in decimal number of years) as covariate to check for the influence of age on overall performances. Since we did not find any effect nor interaction given by the age, follow-up ANOVAs, conducted only for the significant interactions, were run with permutation tests [*aovp()* function using the R package “lmPerm” (Wheeler and Torchiano, 2016)]. The lmPerm package use permutation tests to obtain *p*-values for linear models when data do not follow a normal distribution (Wheeler, 2010). In reporting the results of non-normally distributed data, permutation test *p*-values are reported. Finally, *post hoc* analyses were run with two-tailed unpaired Student’s *t*-tests based on permutations (*perm.t.test()* R function). Effect sizes were calculated in terms of partial eta-squared (η^2^) for ANCOVAs (η^2^: small, > = 0.01; medium, > = 0.06; large, > = 0.14) and as Cohen’s *d* value for the *t*-tests (small, > = 0.2; medium, > = 0.5; large, > = 0.8). Bonferroni correction was used to test the significance of multiple comparison *post hoc* tests (*p* < 0.05 was considered significant).

## Data Availability Statement

The raw data supporting the conclusions of this article will be made available by the authors, without undue reservation.

## Ethics Statement

The studies involving human participants were reviewed and approved by the Comitato Etico, ASL 3, Genoa, Italy. Written informed consent to participate in this study was provided by the participants’ legal guardian/next of kin.

## Author Contributions

WS, LC, and MG conceived the studies and designed the experiments. EC helped in the recruitment of blind participants. All authors wrote and reviewed the manuscript.

## Conflict of Interest

The authors declare that the research was conducted in the absence of any commercial or financial relationships that could be construed as a potential conflict of interest.

## Publisher’s Note

All claims expressed in this article are solely those of the authors and do not necessarily represent those of their affiliated organizations, or those of the publisher, the editors and the reviewers. Any product that may be evaluated in this article, or claim that may be made by its manufacturer, is not guaranteed or endorsed by the publisher.

## Footnotes

1
https://freesound.org/
